# Second Annual Convention of the North Carolina State Dental Society

**Published:** 1858-04

**Authors:** E. H. Andrews, D. W. C. Benbow

**Affiliations:** President; Secretary


### PROCEEDINGS OF SOCIETIES.

### ARTICLE VIII.

### *Second Annual Convention of the North Carolina State Dental Society.*

GREENSBORO', October 7th, 1857.

THE Second Annual Convention of the North Carolina State Dental Society met in this place, agreeable to adjournment.

The president, Dr. Bason, being absent, Dr. Bessent took the chair. Drs. E. H. Andrews and J. H. Wayte of Charlotte, Hubbard of Newbern, and Tate of Statesville, were unanimously elected members.

The next business being that of reading the essays from Dr. Bessent, subject, Sensitive Dentine—Dr. Gregg, subject, Insertion of Artificial Dentures. Dr. Bason being unable to attend, favored us with an interesting and congratulatory epistle, in which he offered the following resolutions, which were unanimously adopted, viz.

*Resolved*, That this Society, anxious for the elevation and approbation of correct principles in our colleges, discountenances the practice of selling office rights to our candidates for graduation who have paid the full ticket to all the Professors, as well calculated to lower their standing with the public and place them beneath the sagacity of dealers in office rights.

*Resolved, further*, That we discountenance the practice of peddling or selling office rights by those properly entitled by graduation to all the privileges and immunities of the profession, to any member of this Society.

*Resolved, further*, That we will mutually aid and assist each other in the dissemination of everything new and interesting which we may in any way obtain possession of after this time, which may have a tendency to advance the interests and honor of this association.

Adjourned to meet next day at ten o'clock.

First business being to elect officers, which resulted as follows:—Dr. E. H. Andrews, President; Dr. D. P. Gregg, Vice-President; Dr. D. W. C. Benbow, Secretary; Dr. Hubbard, Treasurer.

Drs. Scott, Gregg and Benbow, Examining Committee, through whom candidates for admission into the Society will have their names offered at the next Convention.

It was then *Resolved*, That this Society heartily approves of the suggestion made by the National Convention concerning the securing of a chemist or some competent person to conduct experiments, physiological, pathological, chemical and hygienic, as connected with dental science.

We then had a very interesting discussion upon the following subjects, viz. Sensitive Dentine, Filling Nerve Cavities, Abscesses, Fractures, Deformatives, &c., and their best treatment.

Dr. Andrews related the circumstances of a case that came under his charge: it was the mouth of a young man for whom he had, within the last two months, removed eleven teeth from the space that one of the central insisors usually occupies; and that the young man stated prior to the 1st of August he had had from time to time some eight or ten extracted from that same place. Strange freak of nature, that so soon as one tooth becomes loose and is removed, another supplies its place.

Adjourned to evening.

*Met.*—Drs. Andrews, Howlett, Scott, Tate and Benbow were requested to prepare papers for next meeting. Professional fees were agreed upon. Time and place of next meeting being Charlotte, on the last Wednesday in September, 1858.

Drs. Gregg, Scott, Bessent, Andrews, and Benbow, are the delegates to the next annual meeting of the National Convention.

The motion of adjournment was then carried.

E. H. ANDREWS, President.

D. W. C. BENBOW, Secretary.

